# Pregnenolone sulphate-independent inhibition of TRPM3 channels by progesterone

**DOI:** 10.1016/j.ceca.2011.09.005

**Published:** 2012-01

**Authors:** Yasser Majeed, Sarka Tumova, Ben L. Green, Victoria A.L. Seymour, Daniel M. Woods, Anil K. Agarwal, Jacqueline Naylor, Shannon Jiang, Helen M. Picton, Karen E. Porter, David J. O’Regan, Katsuhiko Muraki, Colin W.G. Fishwick, David J. Beech

**Affiliations:** a Multidisciplinary Cardiovascular Research Centre, University of Leeds, Leeds LS2 9JT, UK; b Institute of Membrane & Systems Biology, Faculty of Biological Sciences Health, University of Leeds, Leeds LS2 9JT, UK; c Leeds Institute of Genetics, Health and Therapeutics, Faculty of Medicine & Health, University of Leeds, Leeds LS2 9JT, UK; d School of Chemistry, University of Leeds, Leeds LS2 9JT, UK; e Department of Cardiac Surgery, Leeds General Infirmary, Great George Street, Leeds LS1 3EX, UK; f School of Pharmacy, Aichi-Gakuin University, 1-100 Kusumoto, Chikusa, Nagoya 464-8650, Japan

**Keywords:** Aldo, aldosterone, Cort, cortisol, DhT, dihydrotestosterone, HEK 293 cells, human embryonic kidney cells, La^3+^, lanthanum, Mfp, mifepristone, Nif, nifedipine, PregS, pregnenolone sulphate, Prog, progesterone, 5β, pregnanolone, 5α, allopregnanolone, 17-OH, 17-hydroxyprogesterone, 21-OH, 21-hydroxyprogesterone, Oest, 17β-oestradiol, TG, thapsigargin, TRP, transient receptor potential, VSMC, vascular smooth muscle cell, 2-CMNPBC, 2-chloro-4-(methylsulphonyl)-N-[4-(1 pyrrolidinyl)phenyl]benzenecarboxamide, Cationic channel, Calcium influx, Transient receptor potential, Steroid, Pregnenolone, Progesterone

## Abstract

Transient Receptor Potential Melastatin 3 (TRPM3) is a widely expressed calcium-permeable non-selective cation channel that is stimulated by high concentrations of nifedipine or by physiological steroids that include pregnenolone sulphate. Here we sought to identify steroids that inhibit TRPM3. Channel activity was studied using calcium-measurement and patch-clamp techniques. Progesterone (0.01–10 μM) suppressed TRPM3 activity evoked by pregnenolone sulphate. Progesterone metabolites and 17β-oestradiol were also inhibitory but the effects were relatively small. Dihydrotestosterone was an inhibitor at concentrations higher than 1 μM. Corticosteroids lacked effect. Overlay assays indicated that pregnenolone sulphate, progesterone and dihydrotestosterone bound to TRPM3. In contrast to dihydrotestosterone, progesterone inhibited nifedipine-evoked TRPM3 activity or activity in the absence of an exogenous activator, suggesting a pregnenolone sulphate-independent mechanism of action. Dihydrotestosterone, like a non-steroid look-alike compound, acted as a competitive antagonist at the pregnenolone sulphate binding site. Progesterone inhibited endogenous TRPM3 in vascular smooth muscle cells. Relevance of TRPM3 or the progesterone effect to ovarian cells, which have been suggested to express TRPM3, was not identified. The data further define a chemical framework for competition with pregnenolone sulphate at TRPM3 and expand knowledge of steroid interactions with TRPM3, suggesting direct steroid binding and pregnenolone sulphate-independent inhibition by progesterone.

## 1 Introduction

The Transient Receptor Potential Melastatin 3 (TRPM3) cationic channel is expressed quite widely in mammalian cells such as pancreatic β-cells, oligodendrocytes, vascular smooth muscle cells, and sensory neurones [1–4]. It has been found to modulate secretion of factors including insulin and interleukin-6, promote vascular contraction, enable heat-sensing, and confer zinc influx [3–5] but there remains relatively little knowledge of its physiological significance and regulation. There has been interest in the potential for chemical modulation because many other types of TRP channel serve chemical-sensing functions [6]. TRPM3 has been found to be stimulated, inhibited or unaffected by sphingosine [1,3,7] and stimulated by pregnenolone sulphate [1]. The pregnenolone sulphate effect is most striking, reflecting non-genomic steroid-sensing capability of TRPM3 channels [1]. The data are consistent with the existence of an extracellular stereo-specific binding site, on or near TRPM3, which accepts a limited number of steroids including pregnenolone, pregnenolone sulphate and epipregnanolone sulphate [8]. Many other steroids, including sex steroids, are not stimulators of TRPM3 [1]. Low-level constitutive activity of TRPM3 has been described for over-expressed and endogenous channels [3,9]. An identified endogenous inhibitor is cholesterol, which is the precursor of steroid hormones [3]. Non-physiological chemical modulators of TRPM3 include the dihydropyridine nifedipine which stimulates TRPM3 [1,10], and 2-aminoethoxydiphenyl borate, mefenamic acid and the thiazolidinediones rosiglitazone, troglitazone and pioglitazone which inhibit TRPM3 [10–12]. The aims of this study were to further characterise the steroid sensitivity of TRPM3 and determine if any endogenous steroid hormones are inhibitors of the channel.

## 2 Methods

### 2.1 HEK 293 cell culture

The tetracycline-regulated expression of human TRPM3 in HEK 293 cells has been previously described [8]. Cells not induced with tetracycline (Tet− cells) were controls. The tetracycline-regulated expression of human TRPM2 in HEK 293 has also been described [13]. Cells were maintained in Dulbecco's Modified Eagle's Medium-F12 + GLUTAMAX (Cat # 31331, Gibco, Paisley, UK) supplemented with 10% foetal calf serum, 100 units/ml penicillin/streptomycin (Sigma) and selection antibiotics (10 μg/ml blasticidin and 400 μg/ml zeocin; Invitrogen) at 37 °C in a 5% CO_2_ incubator.

### 2.2 Vascular smooth muscle cells

Freshly discarded human saphenous vein segments were obtained anonymously and with informed consent from patients undergoing open heart surgery in the General Infirmary at Leeds. Approval was granted by the Leeds Teaching Hospitals Local Research Ethics Committee. Proliferating vascular smooth muscle cells (VSMCs) were prepared using an explant technique and grown in Dulbecco's Modified Eagle's Medium + GLUTAMAX (Cat # 31966, GIBCO). The medium was supplemented with 10% foetal calf serum, 100 U/ml penicillin/streptomycin (Sigma) at 37 °C in a 5% CO_2_ incubator. Experiments were performed on cells passaged 3–5 times.

### 2.3 Ovarian granulosa cells

Bovine granulosa cells were harvested from abattoir-derived bovine ovaries from at least 6 heifers at random stages of the oestrous cycle on 5 separate occasions. Reproductive tracts were recovered within 3 h of slaughter and transported to the laboratory at ambient temperature in an insulated box. The ovaries were removed from the reproductive tract and washed in phosphate-buffered saline (PBS, pH 7.4), containing 100 IU/ml penicillin G, 100 μg/ml streptomycin sulphate and 250 ng/ml amphotericin B, which had been pre-warmed to 39 °C. Granulosa cells were isolated using methodology adapted from Gutierrez et al. [14]. During isolation the ovaries were retained in pre-warmed bicarbonate-buffered McCoy's 5a medium supplemented with: 20 mM HEPES, 0.1% (w/v) fatty acid-free bovine serum albumen, 100 IU/ml penicillin G, 100 μg/ml streptomycin sulphate, 2.5 μg/ml bovine transferrin, 4 ng/ml sodium selenite, and 3 mM l-glutamine. 15–20 follicles of 7–20 mm diameter were collected, washed in culture medium, the follicular fluid aspirated using a 1 ml syringe fitted with a 23-gauge needle and each follicle was bisected into a small Petri dish containing 3 ml of cell culture media. The granulosa cells were removed by gently scraping the follicle wall with a sterile inoculation loop and cells were pooled across all follicles. The cell suspension was transferred to a 15-ml centrifuge tube and washed twice in a total volume of 12 ml of medium followed by centrifugation at 1200 rpm for 10 min at room temperature. The cell pellet was re-suspended in 5 ml of culture medium supplemented with 10 ng/ml of bovine insulin, 1 ng/ml of the synthetic insulin like growth factor I (IGF-I) analogue LR3-IGF-I, and ovine follicle stimulating hormone (1 mU/ml) and ovine luteinizing hormone (0.5 mU/ml). At these concentrations, the gonadotrophins induce granulosa cell luteinization and high levels of progesterone production in vitro. The granulosa cell culture media was pre-equilibrated to 39 °C and 5% CO_2_ in air in a humidified incubator. Whole-cell patch-clamp was performed on the day of isolation over a period of 4–5 h.

### 2.4 Ca^2+^ measurement

For intracellular Ca^2+^ measurement, cells were plated at a confluence of 80–90% in clear-bottom poly-d-lysine coated 96-well plates (Corning, NY, USA) for 24 h prior to experiments. Immediately prior to recordings, cells were incubated for 1 h at 37 °C in standard bath solution (SBS) containing 2 μM fura-2 acetoxymethyl ester (and 0.01% pluronic acid) and then washed with SBS once before adding the recording buffer (SBS with appropriate solvent). All experiments were performed at 23 ± 2 °C. SBS contained (mM): NaCl 130, KCl 5, MgCl_2_ 1.2, CaCl_2_ 1.5, glucose 8, d-mannitol 11, and HEPES 10 in deionised water; pH was titrated to 7.4 using 4 M NaOH. Measurements were made on a 96-well fluorescence plate reader (FlexStation II^384^, Molecular Devices, Sunnyvale, CA, USA). Fura-2 was excited by light of 340 and 380 nm and emitted light was collected at 510 nm. Wells of the 96-well plate were studied in a column format and loaded alternately for test and control conditions. Change (Δ) in intracellular calcium (Ca_i_^2+^) concentration is indicated as the ratio of fura-2 fluorescence (*F*) emission intensities for 340 nm and 380 nm (*F* ratio). All experiments were performed at room temperature (23 ± 2 °C), unless indicated otherwise.

### 2.5 Electrophysiology

Borosilicate glass capillaries with an outside diameter of 1 mm and an inside diameter of 0.58 mm (Harvard Apparatus, Holliston, MA, USA) were used as the basis for patch pipettes. Pipette resistance after fire-polishing and when filled with ionic solutions were 3–5 MΩ. The pipettes were mounted on a CV203BU head-stage (Molecular Devices, Sunnyvale, CA, USA) connected to a 3-way coarse manipulator and a micro-manipulator (Newport 300P, Newport Corporation, Irvine, CA, USA). Electrodes comprised silver wires coated with chloride ions. Electrical signals were amplified and recorded using an Axopatch 200B amplifier and pCLAMP 8 software (Molecular Devices, Sunnyvale, CA, USA). Data were filtered at 1 kHz and sampled digitally at 2 kHz via a Digidata 1322A analogue to digital converter (Molecular Devices, Sunnyvale, CA, USA). Recordings were made at least 5 min after break-through to the whole-cell configuration. Series resistance was <10 MΩ. The voltage-ramp protocol was a step from a holding potential of 0 mV to −100 mV, followed by a 0.1-s ramp to +100 mV, before returning to 0 mV (repeated every 10 s). The extracellular solution contained (mM): NaCl 130, KCl 5, CsCl 10, MgCl_2_ 1.2, CaCl_2_ 1.5, glucose 8 and HEPES 10, with pH titrated to 7.4 using 4 M NaOH. The osmolality of this solution was 295 mOsm/L. The patch pipette solution contained (mM): CsAspartate 80, CsCl 45, HEPES 10, BAPTA sodium 10, Na_2_ATP 4; osmolality was adjusted to 290 mOsm/L using d-mannitol and the pH was titrated to 7.2 using 4 M CsOH. The pipette solution was filtered using a 0.2-μm membrane filter (Minisart Sartorius Stedim biotech, Goettingen, Germany), divided into aliquots of approximately 50 μL and stored at −20 °C. All experiments were at 23 ± 2 °C. For K^+^-current recording, the patch pipette solution contained (mM): NaCl 5, KCl 130, MgCl_2_ 2, EGTA 5, HEPES 10, Na_2_ATP 3 (pH 7.4 using KOH). Analysis was performed off-line using Clampfit 8.2 (Molecular Devices, Sunnyvale, CA, USA) and Origin 7.5 software (OriginLab, Northampton, MA, USA). Background-subtracted TRPM3 *I*–*V*s were smoothed using the adjacent-averaging algorithm in Origin 7.5.

### 2.6 Steroid overlay assay

The assay was performed largely as described previously [15]. Briefly, HEK 293 cells transfected with plasmids encoding TRPM3-YFP or GFP were harvested in PBS. Cell pellets were homogenized using 1 ml of 50 mM Tris (pH 7.4) containing 0.2% CHAPS and protease inhibitor cocktail (Fermentas, UK), incubated for 2 h at 4 °C and clarified by centrifugation at 18,000 × *g* at 4 °C for 20 min. The supernatant was diluted (1:5) using 2% fatty acid-free BSA in TNC buffer (50 mM Tris, pH 7.4, 150 mM NaCl, 0.2% CHAPS) and incubated overnight at 4 °C with a PVDF membrane. The PVDF membrane was prepared by spotting different test chemicals (30–60 nmol) dissolved in chloroform/methanol mixture (2:1) and blocking in 10% milk, 2% fatty acid-free BSA in TNC buffer for 2 h. After application of the lysate, the membranes were washed and incubated with mouse anti-GFP antibody (1:6000; Abcam, Cambridge, UK) in 2% fatty acid-free BSA, TNC for 2 h at room temperature. An anti-mouse secondary antibody conjugated to HRP (1:10,000 dilution; Santa Cruz Biotechnology, Inc, Santa Cruz, USA) and Pierce SuperSignal West Femto Substrate (Thermo Fisher Scientific) were used to detect specific binding after brief (5–10 s) exposure to an X-ray film.

### 2.7 Chemicals and reagents

Steroids were purchased from Sigma or Steraloids and stock solutions were stored according to the suppliers’ instructions. The following steroids were prepared as 5–100 mM stocks in 100% DMSO: pregnenolone sulphate, progesterone, 17-hydroxy progesterone and 21-hydroxy progesterone, pregnanolone, allopregnanolone, mifepristone, and nifedipine. The following steroids were prepared as 10–50 mM stocks in 100% ethanol: 17β-oestradiol, dihydrotestosterone, aldosterone and cortisol. Nifedipine (50 mM) was prepared in 100% DMSO. Thapsigargin (Sigma) was prepared as a 5 mM stock in 100% DMSO. 2-Chloro-4-(methylsulphonyl)-N-[4-(1 pyrrolidinyl)phenyl]benzenecarboxamide (2-CMNPBC, Key Organics) was prepared as a 10 mM stock in 100% DMSO. Lanthanum chloride (100 mM) was prepared in deionised water.

### 2.8 Data analysis

Origin 7.5 software (OriginLab Corporation) was used for data analysis and presentation. Averaged data are expressed as mean ± standard error of the mean (SEM). The amplitudes of signals were measured at the peak response. Matched groups of control and test data were either compared using an independent *t*-test (intracellular Ca^2+^ measurement) or paired *t*-test (electrophysiology performed on the same cell). Each test data set had its own control data set. Probability (*P*) of less than 0.05 was considered statistically significant (*); not significant (n.s.) indicates *P* > 0.05. All intracellular Ca^2+^ measurement data are presented as *N*/*n*, where ‘*N*’ is the number of wells used in the 96-well plate and ‘*n*’ is the total number of independent experiments (i.e. on different 96-well plates). For patch-clamp recordings, the number of independent recordings is indicated by ‘*n*’.

## 3 Results

### 3.1 Inhibition of pregnenolone sulphate responses by progesterone

**Fig. 1:**
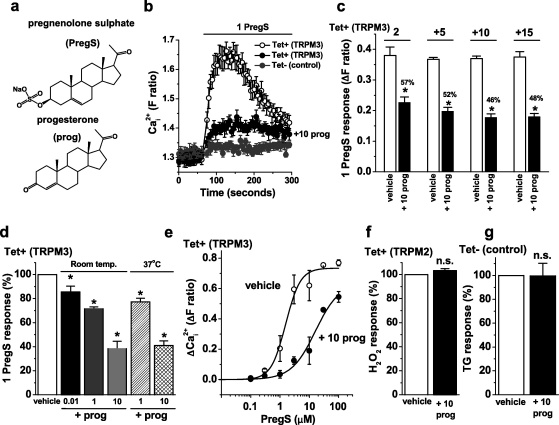
Inhibition of TRPM3-mediated Ca^2+^ influx by progesterone. Data were generated by intracellular Ca^2+^ measurement. (a) Structures of pregnenolone sulphate (PregS) and progesterone (prog). (b) Example responses to 1 μM PregS in cells induced to express TRPM3 (Tet+) in the presence of vehicle (open circles) or 10 μM prog (filled black circles). Control (Tet−) cell data (filled gray circles) are shown for comparison (no response to PregS) (*N* = 8 for each). (c) Cells were treated with 10 μM progesterone and its effect on PregS-activated TRPM3 was determined after 2 min (labelled ‘2’) and at 5 min intervals thereafter. For every time-point, the response in the presence of progesterone is indicated as a percentage of the control response (*N*/*n* = 32/4). (d) Mean normalised data for the effect of different concentrations of prog (in μM) at room temperature (Room temp., *N*/*n* = 24/3) or at 37 °C (*N*/*n* = 22/3). (e) Concentration–response curves for PregS in the absence (vehicle) or presence of 10 μM prog (+10 prog). The fitted Hill equation gave EC_50_ and slope values of 1.56 μM and 1.74 (vehicle) and 16.1 μM and 1.06 (prog), respectively (*N*/*n* = 6/3 for each concentration of PregS). (f) Mean TRPM2 activity stimulated by 1 mM hydrogen peroxide, comparing vehicle (control) with 10 μM prog (*N*/*n* = 24/3 each). (g) Mean 1 μM thapsigargin (TG) responses in non-induced (Tet−) cells in Ca^2+^-free SBS, comparing vehicle (control) with 10 μM prog (*N*/*n* = 24/3 each).

TRPM3 is a Ca^2+^-permeable ion channel and so we used an intracellular Ca^2+^ indicator to measure TRPM3-dependent Ca^2+^-entry in HEK 293 cells. The chemical structures of pregnenolone sulphate and progesterone are similar (Fig. 1a) but pregnenolone sulphate stimulated TRPM3-dependent Ca^2+^-entry whereas progesterone did not (Fig. 1b). HEK 293 cells that were not induced to express exogenous TRPM3 failed to respond to pregnenolone sulphate, demonstrating the TRPM3-dependence of the response (Fig. 1b; Supplementary Fig. I). The lack of stimulatory effect of progesterone has been reported previously [8] but is also evident in Fig. 1b because the baseline Ca^2+^ signal prior to application of pregnenolone sulphate was unchanged despite the fact that progesterone had been pre-incubated with the cells for 15 min and maintained throughout the recordings. A novel observation was that the presence of progesterone inhibited the subsequent response to pregnenolone sulphate (Fig. 1b) within 2–5 min (Fig. 1c). This effect of progesterone occurred at concentrations ranging from 10 nM to 10 μM (Fig. 1d). The effect was similar at room temperature (23 ± 2 °C) and 37 °C (Fig. 1d). Progesterone caused a right-shift of the pregnenolone sulphate concentration–response curve and reduced the slope (Fig. 1e). It also appeared to reduce the maximum response to pregnenolone sulphate, although this could not be fully investigated because of steroid solubility limitations (Fig. 1e). Progesterone was not acting non-specifically to suppress Ca^2+^ signals in the cells because it had no effect on the activity of exogenous TRPM2 channels (Fig. 1f) or endogenous Ca^2+^-release signals evoked by thapsigargin (Fig. 1g).

**Fig. 2:**
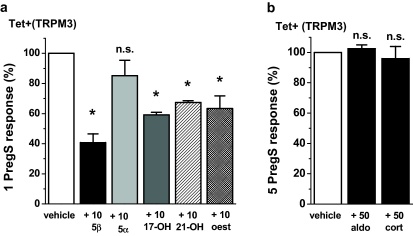
Effects of progesterone metabolites, oestradiol and corticosteroids. Data were generated by intracellular Ca^2+^ measurement in cells over-expressing TRPM3 (Tet+). (a) Mean normalised data for the effect of 10 μM each of pregnanolone (5β), allopregnanolone (5α), 17-hydroxy progesterone (17-OH), 21-hydroxy progesterone (21-OH) or 17β-oestradiol (oest) on responses to 1 μM PregS (*N*/*n* = 24/3 each). (b) As for (a), but tests of 50 μM aldosterone or cortisol on responses to 5 μM PregS (*N*/*n* = 24/3 each).

Progesterone metabolites and 17β-oestradiol also inhibited TRPM3 activity but the effects were equivalent to or smaller than those of progesterone (Fig. 2a, Supplementary Fig. II) and were not investigated further. The progesterone-related steroid allopregnanolone, which is a potent inhibitor of GABA-A channels [16], had no effect on TRPM3 (Fig. 2a). The corticosteroids aldosterone and cortisol also lacked effect (Fig. 2b). Chemical structures of the steroids are shown in Supplementary Fig. III.

**Fig. 3:**
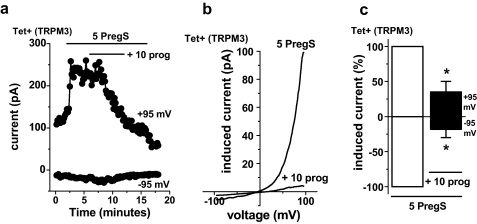
Inhibition of TRPM3-mediated ionic current by progesterone. Data were generated by whole-cell patch-clamp. (a) Example time-series showing the effect of 10 μM prog on current evoked by 5 μM PregS in TRPM3-expressing (Tet+) cells (DMSO vehicle was constant throughout). (b) For the experiment of (a), PregS-evoked *I*–*V* in vehicle (5 PregS) and then plus 10 μM prog (+10 prog). (c) As for (a), but mean currents normalised to pre-prog amplitudes (*n* = 8).

Whole-cell patch-clamp was used as an independent method to measure TRPM3 activity, enabling recordings under voltage-clamp conditions and observation of the shape of the TRPM3 current–voltage relationship (*I*–*V*). As in the Ca^2+^ measurement experiments we used a low concentration of pregnenolone sulphate to stimulate the channels in order to maximise the potential physiological relevance. Therefore, the ionic current evoked by pregnenolone sulphate was relatively small (*cf.* data in Majeed et al. [8]). Over a period of 5 min 10 μM progesterone strongly inhibited ionic current evoked by pregnenolone sulphate (Fig. 3a). The current had the expected current–voltage relationship (*I*–*V*) of TRPM3 (Fig. 3b) [8]. The amplitude of the inhibitory effect was larger than that observed in Ca^2+^ measurement assays, showing almost complete inhibition of the channel activity (Fig. 3c *cf.* Fig. 1d). Progesterone (50 μM) did not cause significantly greater inhibition (*n* = 6, *P* > 0.05, data not shown), suggesting that 10 μM progesterone caused the maximum effect. Recovery from the effect of progesterone was not detected in the 8 min time course of the wash-out period (Fig. 3a).

The data suggest that progesterone is an inhibitor of TRPM3 channels.

### 3.2 Distinctions between effects of progesterone and dihydrotestosterone

**Fig. 4:**
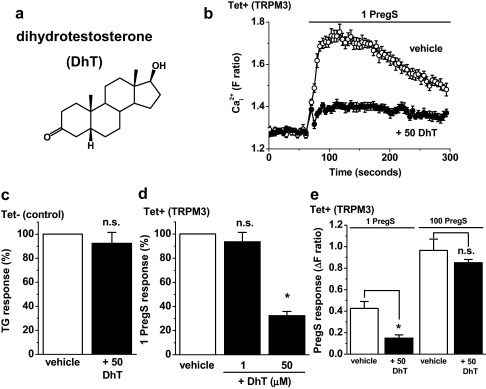
Inhibition of TRPM3 by dihydrotestosterone. Data are from intracellular Ca^2+^ measurements in cells over-expressing TRPM3 (Tet+) (b, d, e) or control (Tet−) cells (c). (a) Structure of dihydrotestosterone (DhT). (b) Example responses to 1 μM PregS in the presence of vehicle or 50 μM DhT (*N* = 8 for each). (c) Mean 1 μM thapsigargin (TG) responses in non-induced (Tet−) cells in Ca^2+^-free SBS, comparing vehicle (control) with 50 μM DhT (*N*/*n* = 24/3 each). (d) Mean normalised data for the effect of DhT at two concentrations on responses to 1 μM PregS (*N*/*n* = 24/3 each). (e) Mean data for the effect of 50 μM DhT on TRPM3-dependent responses elicited by 1 μM or 100 μM PregS (*N*/*n* = 6/3).

**Fig. 5:**
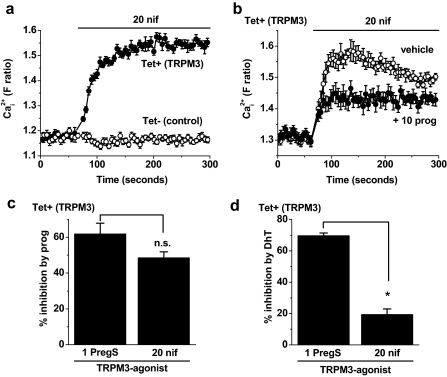
TRPM3 activity evoked by nifedipine. Data are from intracellular Ca^2+^ measurements. (a) Example demonstration of the effect of 20 μM nifedipine (nif) in cells expressing TRPM3 (Tet+) but not control (Tet−) cells. (b) In Tet+ cells, example responses to 20 μM nif in the presence of vehicle or 10 μM progesterone (prog) (*N* = 8 for each). (c), (d) Comparisons of the inhibitory effects of 10 μM prog (c) and 50 μM DhT (d) on TRPM3 stimulated by 1 μM PregS or 20 μM nif (*N*/*n* = 24/3 for each). PregS data are from the same experiments as those underlying Figs. 1d and 4d.

Dihydrotestosterone is chemically similar to progesterone (Fig. 4a *cf.* Fig. 1a). It also inhibited pregnenolone sulphate-evoked Ca^2+^-influx through TRPM3 channels (Fig. 4b). Like progesterone, dihydrotestosterone had no effect on endogenous Ca^2+^ signals evoked by store-depletion with thapsigargin (Fig. 4c). However, in contrast to the effect of progesterone, the effect of dihydrotestosterone occurred only when it was at high concentration: 50 μM and not 1 μM (Fig. 4d). Furthermore, the effect of dihydrotestosterone was surmountable by high concentrations of pregnenolone sulphate (Fig. 4e). These data suggested that there may be a mechanistic difference between the actions of dihydrotestosterone and progesterone. It was hypothesised that dihydrotestosterone competed with pregnenolone sulphate at a binding site while progesterone inhibited TRPM3 via a separate, pregnenolone sulphate-independent, mechanism. To investigate the hypothesis we stimulated TRPM3 using the non-steroidal agent nifedipine [1] (Fig. 5a), the chemical structure of which is given in Supplementary Fig. III. Progesterone inhibited nifedipine-evoked TRPM3 activity (Fig. 5b) and the amplitude of this inhibitory effect was not significantly different from that observed against pregnenolone sulphate-evoked TRPM3 activity (Fig. 5c). In contrast, dihydrotestosterone had a much smaller inhibitory effect against nifedipine-evoked activity compared with pregnenolone sulphate-evoked activity (Fig. 5d). The data suggest that progesterone, in contrast to dihydrotestosterone, could inhibit TRPM3 independently of the presence of pregnenolone sulphate.

### 3.3 TRPM3 activity in the absence of an exogenous agonist

**Fig. 6:**
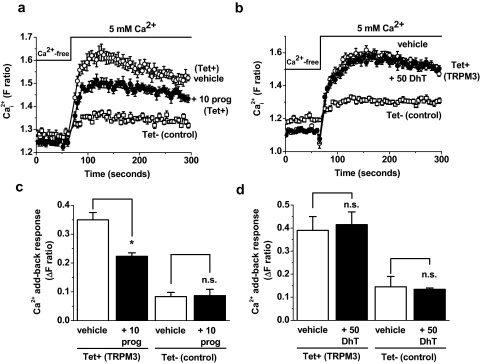
Steroid effects on agonist-independent TRPM3 activity. Data are from intracellular Ca^2+^ measurements. (a), (b) Example traces showing the effects of 10 μM progesterone (a) or 50 μM dihydrotestosterone (b) on the Ca^2+^ signals elicited by addition of 5 mM Ca^2+^ in cells over-expressing TRPM3 (Tet+). Cells were incubated in Ca^2+^-free SBS for 30 min and then Ca^2+^ was ‘added-back’. Responses observed in control (Tet−) cells are shown for comparison (*N* = 8 for each condition). (c), (d) Mean responses to 5 mM Ca^2+^ add-back in the presence of vehicle, 10 μM progesterone (+10 prog) (c) or 50 μM dihydrotestosterone (+50 DhT) (d) (*N*/*n* = 24/3 for each).

Although nifedipine is chemically distinct from pregnenolone sulphate, we could not be sure that it activated TRPM3 independently of the pregnenolone sulphate binding site. Therefore, we also sought TRPM3 activity in the absence of an exogenous agonist. Over-expressed human TRPM3 has been shown to have constitutive activity [9]. However, in our tetracycline-inducible TRPM3 cells (in the presence of 1.5 mM extracellular Ca^2+^) such activity was not evident as a basal Ca^2+^ signal (Fig. 1b; Supplementary Fig. IV) or ionic current (Supplementary Fig. IV). Therefore we used a Ca^2+^ add-back protocol in which 5 mM Ca^2+^ was added to cells after a period in Ca^2+^-free solution (Fig. 6). This protocol revealed a Ca^2+^ response in TRPM3-expressing as well as control cells but the response was significantly larger in the presence of TRPM3 (Supplementary Fig. V). Therefore we used this signal to investigate effects of progesterone and dihydrotestosterone in the absence of an exogenous TRPM3 agonist. Progesterone inhibited the signal in TRPM3-expressing but not control cells (Fig. 6a, c), whereas dihydrotestosterone had no effect in either set of cells (Fig. 6b, d). The data support the hypothesis that progesterone inhibited TRPM3 independently of competition with an exogenous agonist, apparently acting as a mode-independent inhibitor. Dihydrotestosterone, by contrast, had an inhibitory effect that depended on the presence of the agonist pregnenolone sulphate.

### 3.4 Progesterone receptor

**Fig. 7:**
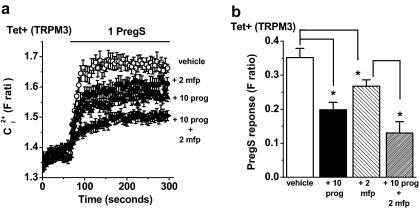
Insensitivity of the progesterone effect to mifepristone. Data were generated by Ca^2+^ measurement in cells over-expressing TRPM3 (Tet+). (a) Cells were treated with 2 μM mifepristone (mfp) or vehicle for 30 min prior to experiments and mfp was maintained during the recordings. Pre-treatment with 10 μM progesterone (prog) was as described in Fig. 1b, and PregS was applied at 1 μM. (b) Mean data for experiments exemplified in (a), showing the effect of prog alone (+10 prog), mfp alone (+2 mfp), or prog with mfp (+10 prog + 2 mfp) on the PregS-induced response (*N*/*n* = 40/5 for each condition).

A mechanism by which progesterone might act on TRPM3 is via a classical progesterone receptor. To investigate this possibility, cells were pre-incubated with the progesterone receptor antagonist mifepristone [17]. At 1–2 μM mifepristone strongly antagonises the progesterone receptor. Mifepristone (2 μM) had an effect of its own on TRPM3-mediated Ca^2+^ influx (24% inhibition) but sufficient TRPM3 activity remained to test progesterone (Fig. 7a, b). The inhibition caused by progesterone in the presence of mifepristone was slightly more than in the absence of mifepristone (52% *cf.* 44% inhibition) (Fig. 7a, b). Mifepristone (1 μM) had no effect of its own on TRPM3 and also did not inhibit the effect of progesterone (data not shown). The data suggest that progesterone did not act on TRPM3 through classical progesterone receptors.

### 3.5 Binding to TRPM3 or a closely associated partner

**Fig. 8:**
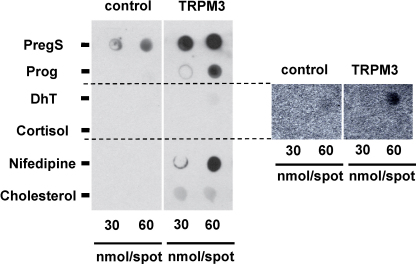
Chemical interaction with TRPM3. Typical result for lysate from HEK 293 cells expressing GFP alone (control) or TRPM3-YFP (TRPM3) incubated with membranes that had been spotted with the indicated chemicals at 30 or 60 nmol (left panel). Detection occurred using anti-GFP antibody. The results for dihydrotestosterone (DhT) and cortisol are duplicated with enhanced contrast in the right panel. The data are representative of three independent experiments.

To investigate whether progesterone or other substances might bind to TRPM3 we performed overlay assays in which cell lysates were applied to membranes spotted with progesterone, pregnenolone sulphate, dihydrotestosterone, cortisol, nifedipine or cholesterol (Fig. 8). Cells were transiently transfected with human TRPM3 tagged with YFP, or GFP-only as a control. Proteins adhering to the spots were detected with anti-GFP antibody, which also binds YFP, a mutant of GFP. TRPM3 bound to pregnenolone sulphate, progesterone, nifedipine, dihydrotestosterone and cholesterol, where as there was no binding to cortisol (Fig. 8). The data are consistent with functional assays in which pregnenolone sulphate, progesterone, nifedipine, dihydrotestosterone and cholesterol have effects on TRPM3 activity but cortisol does not. The data suggest that these TRPM3 modulators act through membrane-delimited mechanisms that involve binding directly to TRPM3, or a partner protein/substance that is tightly bound to TRPM3.

### 3.6 Dihydrotestosterone-like effect of a non-steroidal chemical

**Fig. 9:**
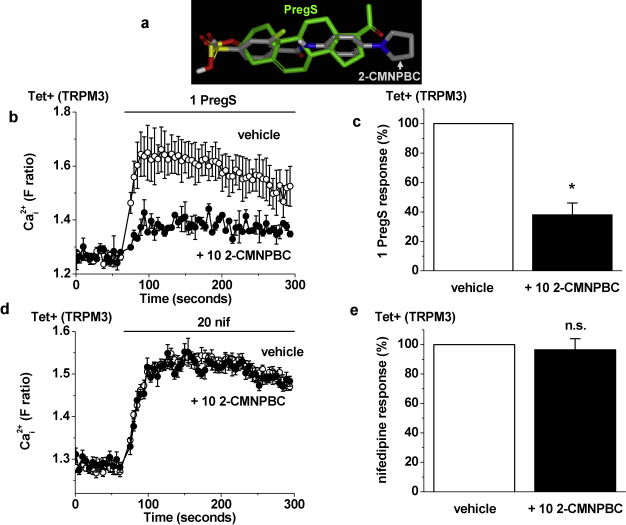
Competition with a non-steroidal look-alike chemical. (a) 3-Dimensional overlay of PregS with the look-alike chemical, 2-CMNPBC. (b), (d) In Tet+ cells, example responses to 1 μM PregS (b) or 20 μM nifedipine (nif) (d) in the presence of vehicle or 10 μM 2-CMNPBC (*N* = 4 for each). (c), (e) Mean data for experiments exemplified in (b) and (d) (*N*/*n* = 12/3).

We previously reported an *in silico* screen of chemical libraries to identify pregnenolone sulphate look-alike compounds that lack the steroid backbone; an inhibitor was identified [8]. Here we show another of these compounds, 2-chloro-4-(methylsulphonyl)-N-[4-(1 pyrrolidinyl)phenyl]benzenecarboxamide (2-CMNPBC, Fig. 9a), which also inhibited pregnenolone sulphate-evoked TRPM3 activity (Fig. 9b, c). Strikingly, the compound had no effect against nifedipine-evoked TRPM3 activity (Fig. 9d, e). Therefore, the look-alike compound mimicked the effect of dihydrotestosterone as an inhibitor of only pregnenolone sulphate-evoked TRPM3 activity.

### 3.7 Endogenous channels

**Fig. 10:**
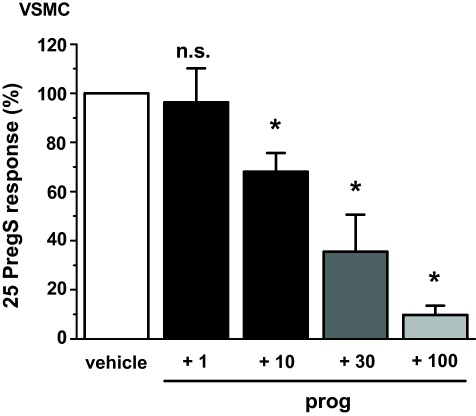
Inhibition by progesterone of Ca^2+^-entry evoked by PregS in vascular smooth muscle cells (VSMCs). Data were generated by intracellular Ca^2+^ measurement from human saphenous vein VSMCs. Shown are mean data for the effect of 15-min treatment with 1, 10, 30 or 100 μM prog on the Ca^2+^ response elicited by 25 μM PregS (*N*/*n* = 9/3 for each concentration).

The above data were generated from HEK 293 cells that conditionally over-expressed human TRPM3. To investigate the relevance to endogenous TRPM3 we measured Ca^2+^-influx evoked by pregnenolone sulphate in human vascular smooth muscle cells (VSMCs), which is mediated at least partly by TRPM3 [3]. Pregnenolone sulphate (25 μM) was used in these experiments because the pregnenolone sulphate concentration–response curve is right-shifted compared with that of over-expressed TRPM3 [3]. Progesterone 10–100 μM inhibited this pregnenolone sulphate-evoked Ca^2+^ signal (Fig. 10). The data suggest that endogenous TRPM3 channels can be inhibited by progesterone.

**Fig. 11:**
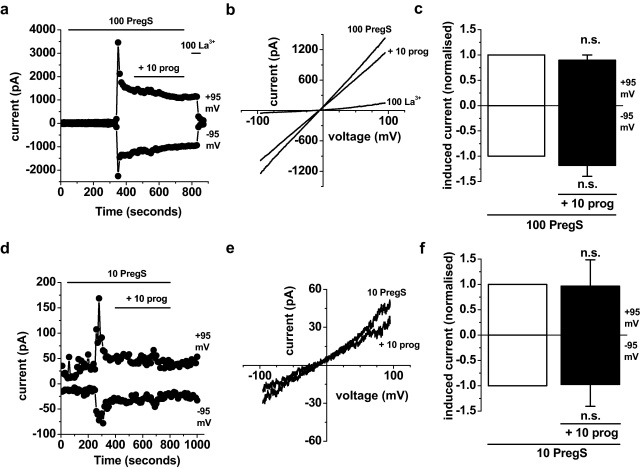
Progesterone resistant ionic currents in granulosa cells. Data were generated by whole-cell patch-clamp performed in freshly isolated bovine granulosa cells. (a) Example time-series plot showing the effect of 10 μM prog on current evoked by 100 μM PregS. 100 μM lanthanum (La^3+^) was applied at the end as a quality control for the whole-cell recording. (b) For the experiment of (a), *I*–*V*s in the presence of 100 μM PregS, with 10 μM prog (+10 prog), or 100 μM La^3+^. (c) As for (a), but mean currents normalised to pre-prog amplitudes (*n* = 4). (d) Example time-series plot showing the effect of 10 μM prog on current evoked by 10 μM PregS. (e) For the experiment of (d), *I*–*V*s in the presence of 10 μM PregS, or with 10 μM prog (+10 prog). (f) As for (d), but mean currents normalised to pre-prog amplitudes (*n* = 5).

We speculated that progesterone regulation of TRPM3 might also be relevant to the female reproductive system where progesterone has potent biological effects. A previous report suggested TRPM3 expression in human ovaries [9]. There were no reports of functional TRPM3 channels in this context and so we investigated if functional channels exist by making whole-cell patch-clamp recordings from granulosa cells freshly isolated from bovine ovarian follicles [14]. In K^+^-current recording solutions, A-type K^+^-current was observed (Supplementary Fig. VI), as expected for this cell type [18]. In TRPM3 recording solutions, pregnenolone sulphate evoked large ionic currents but only after delays of several minutes (Fig. 11a, d), which is not expected for TRPM3 (*cf.* Fig. 3a). The *I*–*V*s of the evoked currents were almost linear (Fig. 11b, e), which is not standard for TRPM3 (*cf.* Fig. 3b), although has been observed in some recordings [19]. Progesterone did not inhibit the currents evoked by pregnenolone sulphate (Fig. 11a–f). The data suggest that granulosa cells contain ionic currents that are stimulated by pregnenolone sulphate but that these currents may not be mediated by TRPM3 and are not inhibited by progesterone.

## 4 Discussion

The study has revealed relatively rapid effects of sex steroids on TRPM3. All effects were inhibitory but there were two distinct mechanisms: one was observed with dihydrotestosterone, the other with progesterone. In the case of dihydrotestosterone, the effect occurred only at high (non-physiological) concentrations and appeared to be due to the steroid competing at a binding site with pregnenolone sulphate. Similar effects were observed with a non-steroid look-alike compound. Progesterone, by contrast, was more potent, showing effects at 10 nM and having maximum effect at 10 μM. Furthermore, a characteristic of progesterone's effect was that it was retained in the absence of pregnenolone sulphate or other exogenous TRPM3 activators, suggesting a general inhibitory effect on all modes of TRPM3 activity. Overlay assays suggested binding of pregnenolone sulphate, progesterone and dihydrotestosterone to TRPM3. The data support the concept of a binding site for pregnenolone sulphate on TRPM3 at which competition occurs (e.g. with dihydrotestosterone or steroid look-alike compounds) but also suggest the existence of a mechanism by which progesterone binds and inhibits TRPM3. Progesterone may bind at or near the pregnenolone sulphate site but its inhibitory effect did not depend on the presence of pregnenolone sulphate.

The findings compare favourably to recent evidence of TRPC5 sensitivity to steroids in which progesterone and dihydrotestosterone were inhibitory [20]. Similar to TRPM3, β-oestradiol and cortisol had less or no effect [20]. Relatively rapid effects of progesterone have also been observed on other types of ion channel, although mostly at relatively high, micromolar, concentrations. Inhibition of voltage-dependent and Ca^2+^-activated K^+^ currents was observed in T cells but there was no effect on Ca^2+^-release-activated Ca^2+^ (CRAC) channel current [21]. P2X channel activity was potentiated by progesterone [22], as was activity of glycine and GABA-A receptors [23]. In the heart, nanomolar progesterone enhanced K^+^-current (*I*_Ks_) under basal conditions and inhibited Ca^2+^ current (*I*_Ca,L_) under cyclic AMP-stimulated conditions; the effects occurred through a PI3-kinase signalling pathway in caveolae [24]. Progesterone stimulated the sperm-specific Ca^2+^-permeable CatSper channel with an EC_50_ in the range of 8–66 nM [25,26]. It has been suggested that special G protein-coupled receptors might mediate non-genomic effects of progesterone [27] but G protein-coupled receptor modulation of TRPM3 channels has not, so far, been identified [3].

Effects of progesterone on TRPM3 were observed at the high end of the physiologically relevant concentration range, which has been observed to be 1–2 nM rising to 30–50 nM in the luteal phase of the menstrual cycle [28,29]. This raises the possibility that TRPM3 is regulated by progesterone *in vivo*. Our observations did not support a role for TRPM3 or its regulation by progesterone in the ovaries, but this is only one aspect of the reproductive system and there may be important differences in relation to TRPM3 in humans, cows and various hormonal contexts. Therefore, further investigation of TRPM3 and its steroid sensitivity is still likely to be worthwhile. It was notable that the endogenous TRPM3 signal in vascular smooth muscle cells was less sensitive to progesterone compared with TRPM3 over-expressed in HEK 293 cells. An explanation for this difference could be that the progesterone mechanism is regulated, perhaps by other types of TRP protein that contribute to the TRPM3-related signal, affecting progesterone sensitivity. It should be considered whether heat is relevant to the steroid sensitivity of TRPM3 because Ca^2+^ events occurred in response to concentrations of pregnenolone sulphate as low as 0.1 μM in 37 °C recordings from HEK 293 cells expressing mouse TRPM3 [4]. Therefore, in the contexts of normal body temperature, fevers or hot flushes, steroids may have greater ability to modulate TRPM3 channels; if there is noxious heat, there may be even stronger effects. Against this hypothesis is our observation that the effect of progesterone was not different at 37 °C (Fig. 1d). Furthermore, we did not observe enhanced potency of pregnenolone sulphate when recording Ca^2+^ entry through human TRPM3 in HEK 293 cells at 37 °C (Supplementary Fig. VII). Our studies have focused on human TRPM3, however. There are substantial species differences in TRPM3 sequence between mouse and human and quite large numbers of splice variants [30]. These differences in sequence may be important in the effects of temperature.

In conclusion, the study provides further insight into the chemical structures that interact with the steroid agonistic binding site associated with TRPM3 channels, supporting the concept of a binding site on or very near to TRPM3 and providing information about interaction with the male sex steroid dihydrotestosterone and non-steroid antagonists. In addition, the study reveals a separate non-genomic steroid effect on TRPM3 that is inhibitory and potentially relevant to physiological progesterone (or other steroids not yet investigated in relation to this mechanism). The inhibitory effect does not depend on the presence of the TRPM3 agonist pregnenolone sulphate although it would be premature to exclude any interaction between the progesterone and pregnenolone sulphate mechanisms. This previously unrecognised steroid effect of progesterone is relatively rapid in its onset but slower than the agonist effect of pregnenolone sulphate.

## Conflict of interest

The authors state no conflict of interest.
