# Supplementary material for: Pregnenolone sulphate-independent inhibition of TRPM3 channels by progesterone
Source: Cell Calcium. 2012 Jan;51(1):1–11. doi: 10.1016/j.ceca.2011.09.005 (PMC3275754; doi:10.1016/j.ceca.2011.09.005)
Supplement: Supplementary file 1 [file mmc1.pdf]

SUPPLEMENTARY INFORMATION

Pregnenolone sulphate-independent inhibition of TRPM3 channels by progesterone

Yasser Majeed[1,2], Sarka Tumova[1,2], Ben L Green[1,2], Victoria AL Seymour[1,2], Daniel M Woods[1,2], Anil K Agarwal[4], Jacqueline Naylor[1,2], Shannon Jiang[1,2], Helen M Picton[1,3], Karen E Porter[1,3], David J O'Regan[5], Katsuhiko Muraki[2,6], Colin WG Fishwick[4] & David J Beech[1,2]*

[1]Multidisciplinary Cardiovascular Research Centre and [2]Institute of Membrane & Systems Biology, Faculty of Biological Sciences and [3]Leeds Institute of Genetics, Health and Therapeutics, Faculty of Medicine & Health, University of Leeds, Leeds, LS2 9JT, UK. [4]School of Chemistry, University of Leeds, Leeds, LS2 9JT. [5]Department of Cardiac Surgery, Leeds General Infirmary, Great George Street, Leeds, LS1 3EX, UK. [6]School of Pharmacy, Aichi-Gakuin University, 1-100 Kusumoto, Chikusa, Nagoya 464-8650, Japan.

*author for correspondence: Professor David J Beech, Institute of Membrane & Systems Biology, Garstang Building, Faculty of Biological Sciences, University of Leeds, Leeds, LS2 9JT, England (UK). Tel: +44-(0)-113-343-4323; Fax: +44-(0)-113-343-4228; Email: d.j.beech@leeds.ac.uk

Supplementary figure legends

Supplementary Figure I. PregS-evoked calcium signals in HEK 293 cells depend on exogenous TRPM3 expression. Mean data from intracellular Ca^{2+} measurement experiments comparing responses evoked by 1 µM PregS in cells over expressing TRPM3 (Tet+), control non-induced (Tet-) cells, or wild-type (WT) HEK 293 cells (N/n = 24-36/3). For example traces, see Supplementary Fig. IV.

Supplementary Figure II. Effects of additional progesterone analogues on TRPM3-mediated Ca^{2+}-influx. Data were generated by intracellular Ca^{2+} measurement. Mean normalised data are shown for the effects of 25 µM each of pregnanolone (5β) or allopregnanolone (5α) on Ca^{2+} responses to 10 µM PregS (N/n = 9/3 for each).

Supplementary Figure III. Additional 2-dimensional chemical structures. Chemical structures of 17-hydroxy progesterone, 21-hydroxy progesterone, pregnanolone, allopregnanolone, 17β-oestradiol, aldosterone, cortisol, and nifedipine.

Supplementary Figure IV. TRPM3 activity in the absence of an exogenous agonist. a, b. Data were generated by intracellular Ca^{2+} measurement. **a.** Example traces showing the effect of 1 µM PregS in cells over-expressing TRPM3 (Tet+), control (Tet-) cells, or wild-type (WT) HEK 293 cells (N = 8-12). **b.** Mean data comparing basal Ca^{2+} levels in the indicated groups (N/n = 24-36/3). **c, d.** Whole-cell voltage-clamp was performed in cells over-expressing TRPM3 (Tet+) or control (Tet-) cells. Statistical comparisons were made between data from these two types of cell. **c.** Mean data for basal currents recorded in cells over-expressing TRPM3 (Tet+) or control (Tet-) cells. **d.** Mean data for the currents stimulated by 100 µM PregS in Tet+ and Tet- cells in the recordings shown in **c** (n = 3).

Supplementary Figure V. Detection of agonist-independent TRPM3 activity. Data were generated by intracellular Ca^{2+} measurement. **a, b.** Cells were incubated in Ca^{2+}-free SBS for 30

min and then Ca^{2+} was returned (“add-back”) at 1, 3 or 5 mM in Tet+ (TRPM3) (**a**) or Tet- (control) (**b**) cells (N=4 for each condition).

Supplementary Figure VI. A-type potassium current in granulosa cells. Example whole-cell patch-clamp recording of an endogenous potassium current in freshly isolated bovine granulosa cells. The voltage paradigm is depicted in the inset.

Supplementary Figure VII. PregS potency at room temperature and 37 °C. Data are from intracellular Ca^{2+} measurements in cells over-expressing TRPM3 (Tet+). Concentration-response curves for PregS at room temperature (Room temp., white circles) or at 37 °C (black circles). The fitted Hill equations had EC_{50} and slope values of: 1.21 μM and 1.34 (Room temp.), and 1.83 μM and 1.19 (37 °C) (N/n = 9/3 for each).

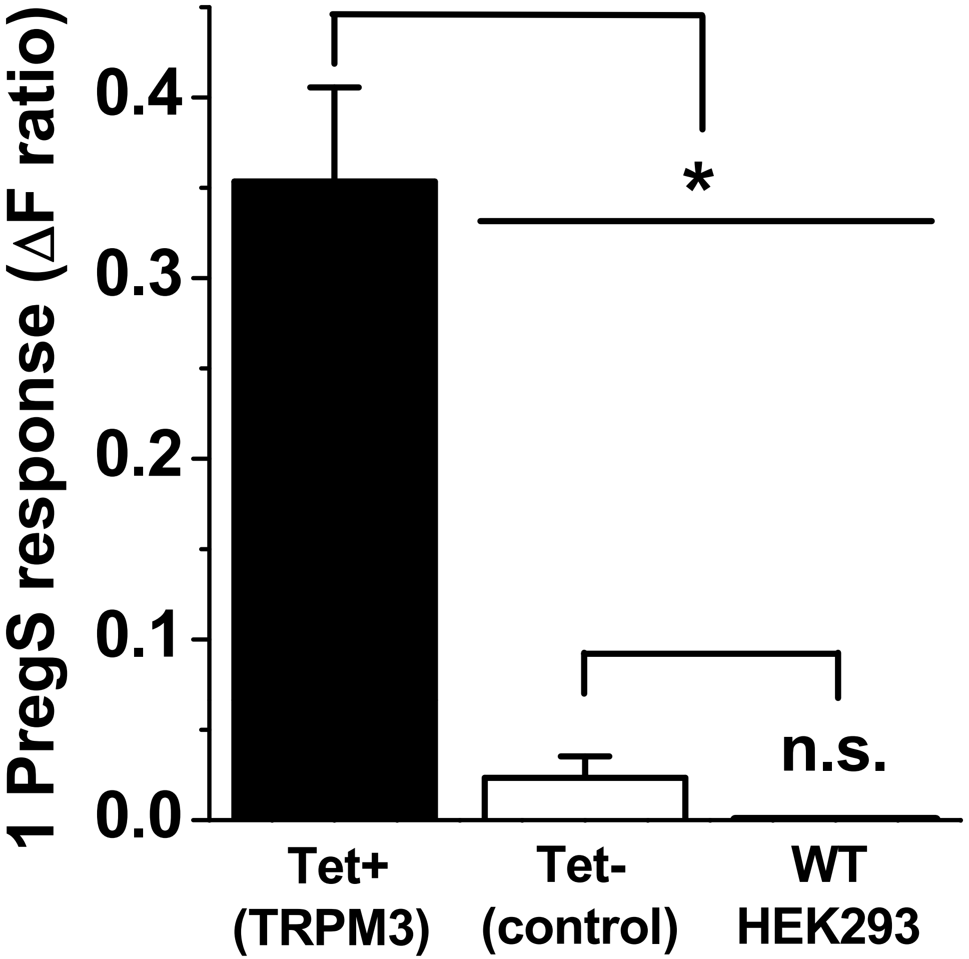

Supplementary figure I

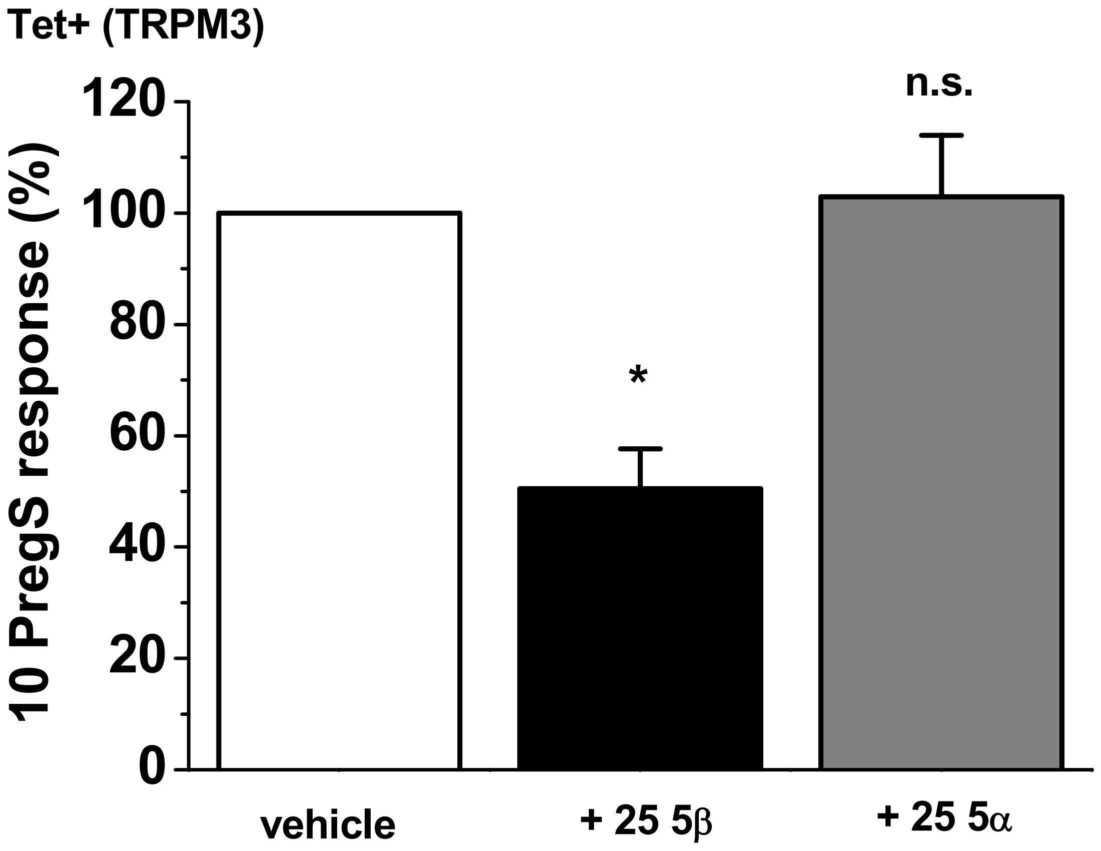

Supplementary figure II

17-OH progesterone

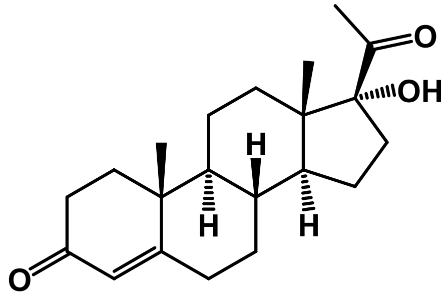

21-OH progesterone

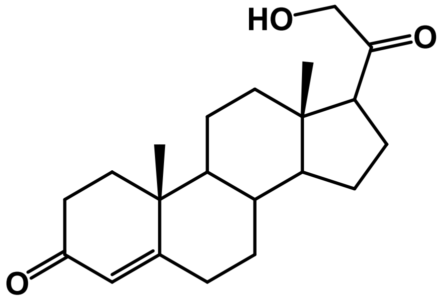

pregnanolone (5β)

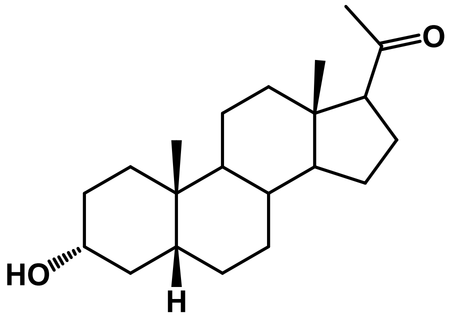

allopregnanolone (5α)

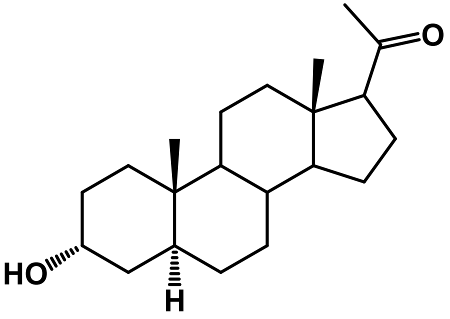

17β-oestradiol

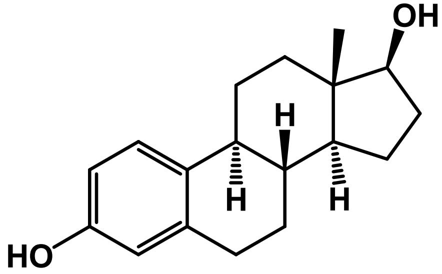

aldosterone

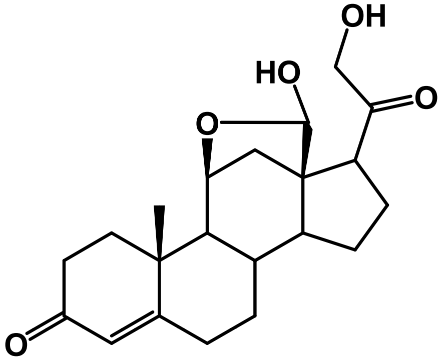

cortisol

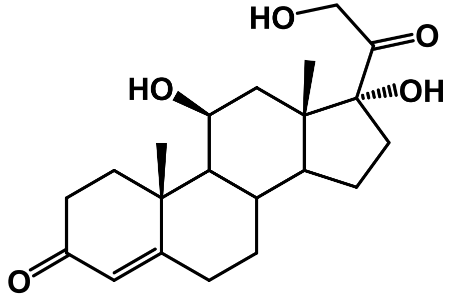

nifedipine

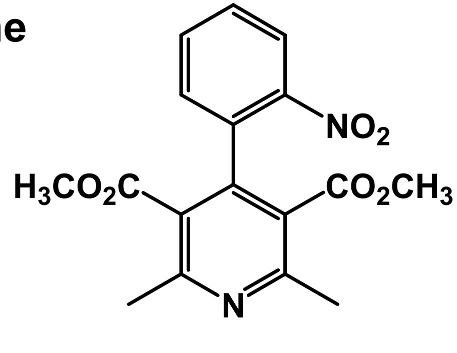

Supplementary figure III

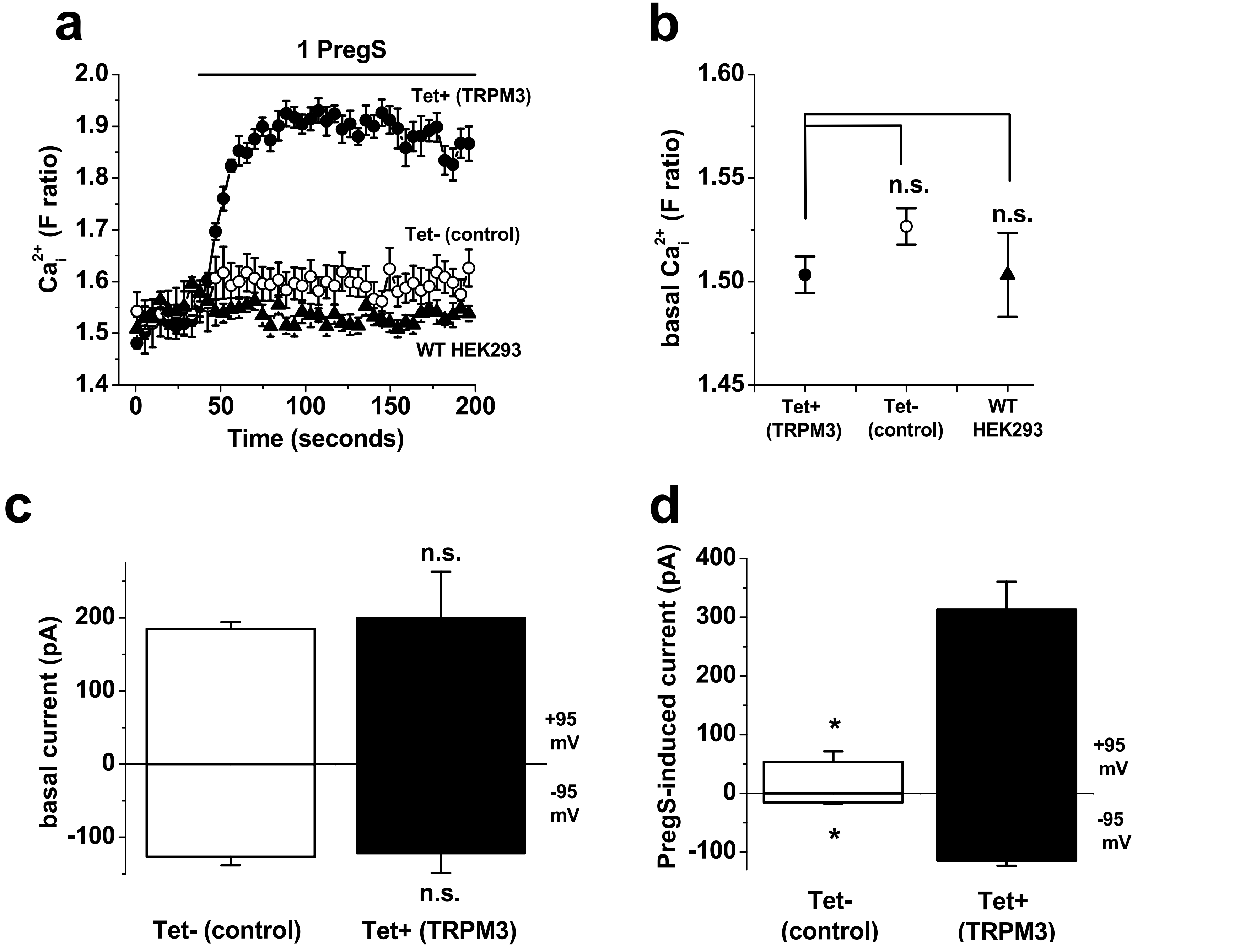

Supplementary figure IV

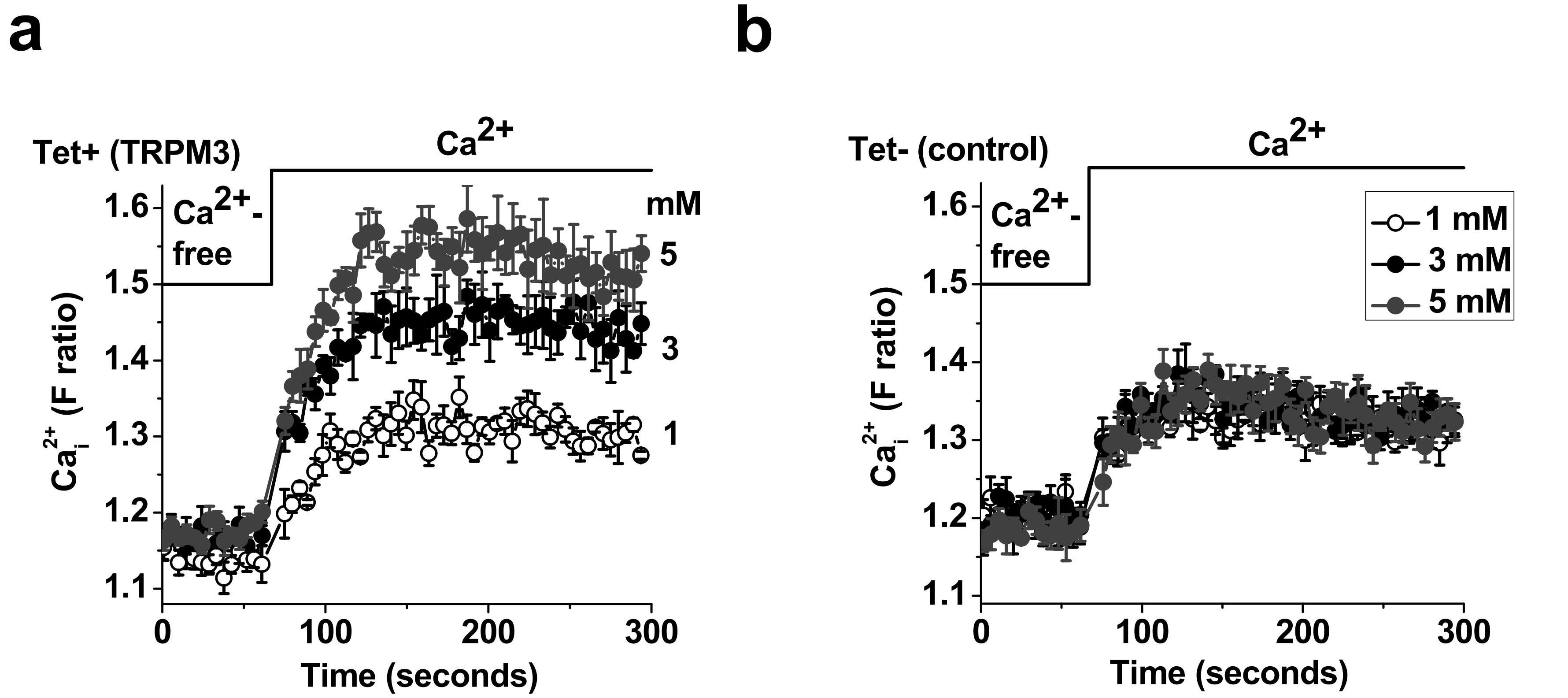

Supplementary figure V

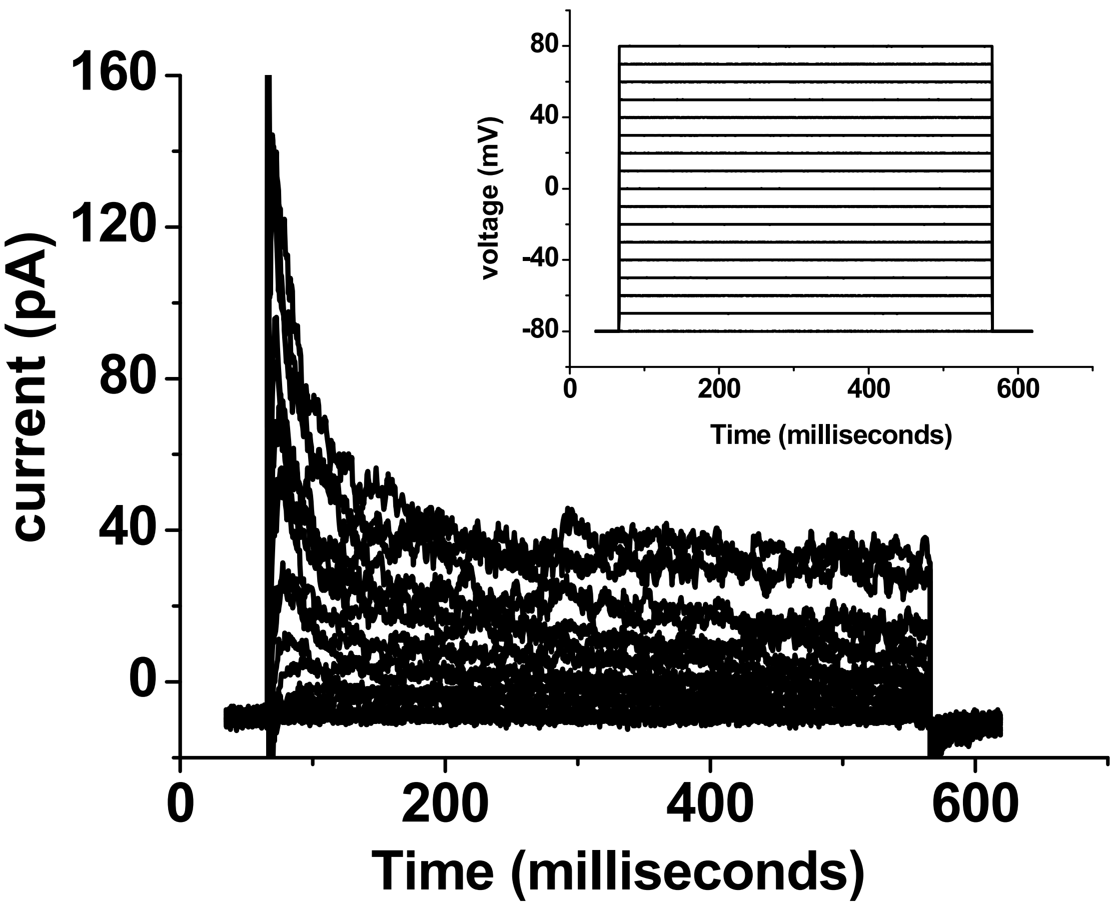

Supplementary figure VI

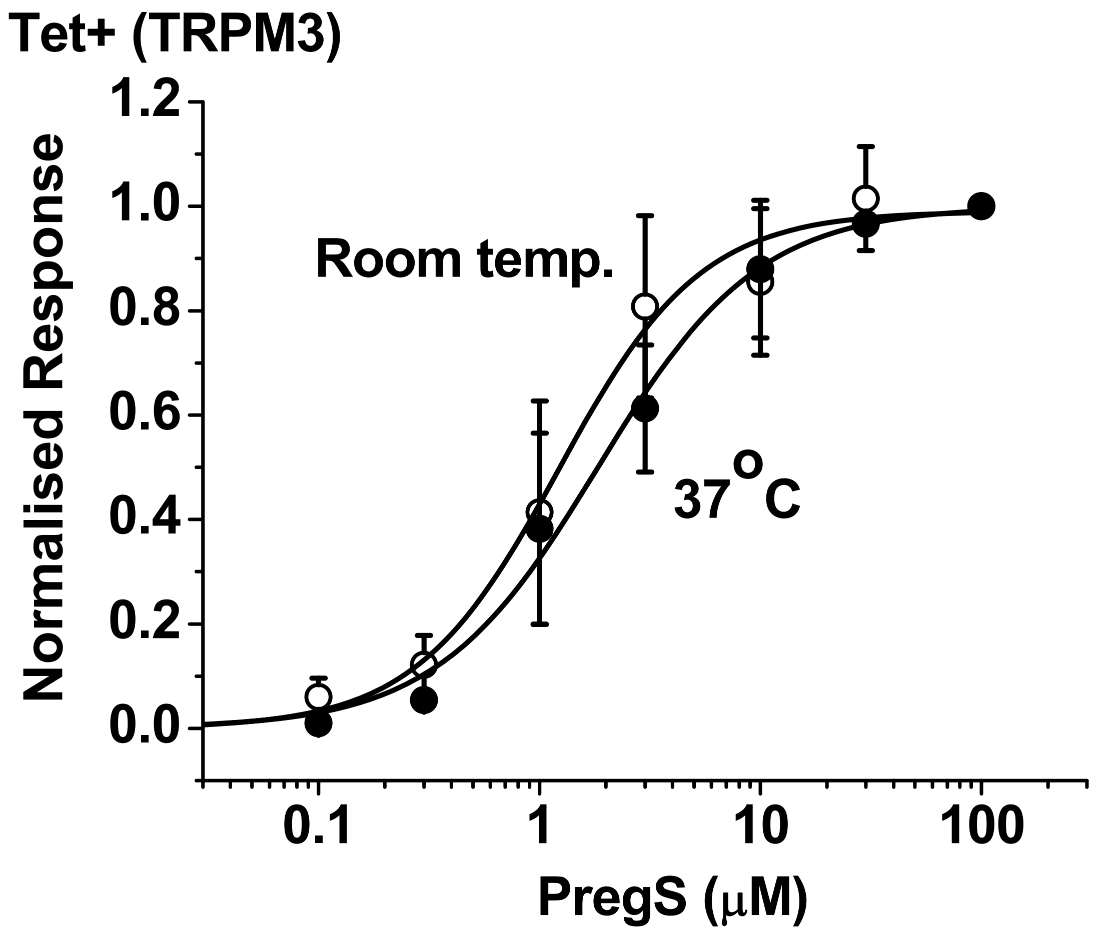

Supplementary figure VII
